# Investigation of endoplasmic reticulum stress-regulated chaperones as biomarkers in idiopathic nonobstructive azoospermia

**DOI:** 10.1016/j.cstres.2024.08.004

**Published:** 2024-09-03

**Authors:** Cigdem Cicek, Pelin Telkoparan-Akillilar, Semra Sertyel, Cumhur Bilgi, Osman Denizhan Ozgun

**Affiliations:** 1 Department of Medical Biochemistry, Faculty of Medicine, Yuksek Ihtisas University, Ankara 06530, Turkey; 2 Department of Medical Biology, Faculty of Medicine, Gazi University, Ankara 06500, Turkey; 3 Department of Medical Biology, Faculty of Medicine, Yuksek Ihtisas University, Ankara 06530, Turkey; 4 Alife Hospital IVF Center, Ankara 06794, Turkey; 5 Alife Hospital Biochemistry Laboratory, Ankara 06794, Turkey

**Keywords:** Male infertility, Idiopathic nonabstructive azoospermia, Endoplasmic reticulum stress, Endoplasmic reticulum chaperones

## Abstract

Azoospermia is a condition in which sperm cells are completely absent in a male's ejaculate. Typically, sperm production occurs in the testes and is regulated by a complex series of cellular and molecular interactions. Endoplasmic reticulum (ER) stress arises when there is a deviation from or damage to the normal functions of the ER within cells. In response to this stress, a cascade of response mechanisms is activated to regulate ER stress within cells. This study aims to investigate the role of ER stress-regulated chaperones as potential biomarkers in male infertility. ER stress associated with azoospermia can manifest in cells such as spermatogonia in the testes and can impact sperm production. As a result of ER stress, the expression and activity of a variety of proteins within cells can be altered. Among these proteins are chaperone proteins that regulate the ER stress response. The sample size was calculated to be a minimum of 36 patients in each group. In this preliminary study, we measured and compared serum levels of protein disulfide-isomerase A1, protein disulfide-isomerase A3 (PDIA3), mesencephalic astrocyte-derived neurotrophic factor (MANF), glucose regulatory protein 78 (GRP78), clusterin (CLU), calreticulin (CRT), and calnexin (CNX) between male subjects with idiopathic nonobstructive azoospermia and a control group of noninfertile males. Serum PDIA1 (*P* = 0.0004), MANF (*P* = 0.018), PDIA3 (*P* < 0.0001), GRP78 (*P* = 0.0027), and CRT (*P* = 0.0009) levels were higher in the infertile group compared to the control. In summary, this study presents novel findings in a cohort of male infertile patients, emphasizing the significance of incorporating diverse biomarkers. It underscores the promising role of ER stress-regulated proteins as potential serum indicators for male infertility. By elucidating the impact of ER stress on spermatogenic cells, the research illuminates the maintenance or disruption of cellular health. A deeper understanding of these results could open the door to novel treatment approaches for reproductive conditions, including azoospermia.

## Introduction

Infertility is defined by the World Health Organization (WHO) as the inability to achieve conception after 12 months or more of uninterrupted sexual activity.^1^, ^2^ Globally, infertility poses a significant health challenge, impacting an estimated 8–12% of couples of reproductive age.^3^ Studies conducted between 1990 and 2017 have demonstrated an annual increase of 0.370% in age-standardized infertility prevalence in women and 0.291% in men.^4^ In 20–30% of cases, male factors are the only cause of infertility; in more than 20% of cases, men are the only responsible.^5^, ^6^ A seminal meta-analysis by Carlsen *et al.*^7^ confirmed a 50% decline in sperm count over six decades. Male infertility is influenced by a variety of factors, many of which remain unclear.^8^ The WHO advises that traditional semen analysis should be the first approach in assessing a man's fertility potential.^9^ Receiver operating characteristic (ROC) curve analysis was utilized in a study by Ombelet *et al.*^10^ to assess the diagnostic usefulness of both individual and combination sperm characteristics. Their findings highlighted that relying on a single sperm parameter has limited effectiveness in distinguishing fertile men from infertile ones, underscoring the necessity of considering multiple sperm parameters to accurately assess fertility status.^10^ However, adherence to WHO manual methods is not consistent across laboratories, with fewer than 60% in the USA and less than 5% in the UK following WHO guidelines.^11^, ^12^ Strict adherence to WHO guidelines by all laboratories is imperative to ensure the reliability and comparability of results. Strict adherence to WHO guidelines by all laboratories is imperative to ensure the reliability and comparability of results. Azoospermia, the most severe form of male infertility, constitutes approximately 10–15% of all infertility cases.^2^, ^13^ Furthermore, certain idiopathic factors contribute to the development of nonobstructive azoospermia.

The folding and transport of secreted and membrane proteins, as well as the metabolism of lipids and carbohydrates and detoxification, depend on the endoplasmic reticulum for cellular production.^14^ Endoplasmic reticulum stress (ERS) is a natural response to adverse external conditions, often triggering inflammatory reactions through various mechanisms to maintain homeostasis or contribute to inflammatory diseases.^15^ The buildup of unfolded or misfolded proteins within the ER lumen is caused by an imbalance between the protein load and the ER's folding ability, which is known as ERS. The unfolded protein response (UPR) is the term used to describe this reaction.^16^, ^17^ ERS is regulated by three sensor proteins: PKR-like ER kinase (PERK), inositol-requiring transmembrane kinase/endoribonuclease (IRE1α), and activating transcription factor 6. Under normal conditions, these proteins associate with glucose regulatory protein 78 (GRP78), remaining inactive. While the mechanism of unfolded protein detection in the ER by PERK and IRE1α remains elusive, Carrara *et al.*^18^ proposed a noncanonical interaction between the ATPase domain of the ER chaperone GRP78 and the luminal domains of UPR sensors IRE1 and PERK, dissociating upon unfolded protein binding to the canonical substrate binding domain of GRP78. Under ER stress, GRP78 is consequently released, which triggers UPR activation *via* further PERK and IRE1α dimerization and autophosphorylation as well as controlled intramembrane proteolysis of activating transcription factor 6. Various resident chaperone proteins within the ER mediate the folding of transmembrane and secreted proteins.^19^ During ERS, ER-resident chaperone proteins are upregulated to facilitate UPR, proper folding, and post-translational modifications of transmembrane and secretory proteins, aiming to restore proteostasis.^20^ These proteins typically contain Lys-Asp-Glu-Leu-like ER membrane-targeting sequences.^21^, ^22^, ^23^ ER homeostasis relies on ER chaperone proteins such as GRP78, GRP94, calreticulin, and protein disulfide isomerase.^24^

Calnexin and CRT, two ER chaperones, have received a lot of interest over the last decade because of their distinct substrate recognition processes, intimate engagement with the Asparagine (Asn)-linked glycosylation system, and different physiological roles.^25^ CNX, a 90 kDa type I ER membrane protein, predominantly resides within the ER lumen,^26^ while CRT, a 60 kDa soluble protein, is localized to the ER lumen.^27^ Both chaperones bind to the majority of glycoproteins traversing the ER, facilitating their correct folding.^28^ These chaperones evolved alongside Asn-dependent glycosylation in eukaryotes, with their preference for glycoproteins arising from their lectin nature, specifically targeting a transient oligosaccharide processing intermediate with a single terminal glucose residue. Clusterin serves as a stress-induced, multifunctional molecular chaperone found both in secreted and cytoplasmic compartments. Cytoplasmic CLU levels rise in response to various ER stressors, including heat shock, proteasome or chaperone (e.g., Hsp90) inhibition, and disruptions of ER homeostasis.^29^, ^30^ Initially identified in ram testicular fluid in 1983,^31^ studies suggest that seminal plasma CLU might serve as a significant biomarker in male infertility.^32^

The catalysis of disulfide (SS) bonds is a defining characteristic of the protein disulfide isomerase (PDI) family. This catalytic process predominantly occurs in the ER, which houses numerous proteins, most of which are naturally secretory and possess at least one disulfide bond. Among the PDI family members, PDIA1^33^ and protein disulfide-isomerase A3 function as chaperones. PDIA1 and PDIA3 exhibit high expression levels in response to cellular stress and play crucial roles in inhibiting ER stress-induced apoptotic cellular death. Within the ER, PDIA1 and PDIA3 oversee the folding of newly synthesized glycoproteins and facilitate the refolding of misfolded proteins, thereby safeguarding cells from ER stress-induced apoptosis. Moreover, these proteins fulfill various functions in the cytosol and nucleus.^34^ PDIA3 interacts with CRT and CNX, two lectin-binding chaperone proteins in the ER, demonstrating greater specificity for glycoproteins than other PDIs.^35^ Furthermore, PDI has chaperone activity that is separate from its disulfide isomerase function, as it binds to misfolded proteins to prevent aggregation.^36^

Fertility researchers are becoming interested in the PDI family, which facilitates the disulfide bond rearrangement required for sperm adhesion proteins to interact with their counterparts in egg cells. The Primakoff group found that PDI inhibitors such as bacitracin could prevent sperm–egg fusion *in vitro*.^37^ They observed the expression of the PDI homolog ERp57 on the sperm surface and noted that antibodies against ERp57 also impede sperm–egg fusion. Visualizing ERp57 on sperm, Ellerman *et al.*^37^ suggested that alternative PDIs, such as ERp29, might aid in priming sperm for fusion. Further investigation utilizing more specific reagents is warranted to comprehensively grasp the redox control of sperm head proteins. Nevertheless, ERp57 was found to be downregulated in a cohort of male *in vitro* fertilization (IVF) patients with low fertilization rates.^38^

The fourth family of neurotrophic factors includes mesencephalic astrocyte-derived neurotrophic factor. MANF is a secreted protein, and ER stress increases its expression and secretion.^39^, ^40^ Moreover, it was found that as the condition worsened, MANF was differentially regulated by a number of ER stress activators and widely expressed in mammalian tissues.^41^ Both neurons and non-neuronal tissues exhibit high levels of MANF messenger RNA (mRNA) and protein expression. Notably, high levels of MANF are seen in the cerebral cortex, hippocampus, and cerebellar Purkinje cells in the brain, but non-neuronal organs such as the adult liver, testis, and salivary gland have higher MANF expression.^41^

In the literature, studies on the role of ER stress-induced chaperones are limited, and the specific biomarker potential of these proteins in male infertility has not yet been fully investigated. Existing studies generally focus on the general biological effects of ER stress and do not specifically investigate its effect on male reproductive health. The original contribution of this study aims to fill an important gap in this field by identifying these specific biomarkers. The innovative aspects of using ER stress-induced chaperone proteins as biomarkers arise from the fact that these proteins can provide information that cannot be obtained by classical sperm analysis methods. This study may provide a new diagnostic tool in cases that cannot be diagnosed by classical methods (such as idiopathic cases). Furthermore, the study of the effects of these chaperones on sperm function through ER stress offers a new perspective in the existing literature. For example, it has been suggested that the presence of high levels of these proteins may indicate a disruption in sperm production in response to cellular stress.

Unlike existing studies, this study identifies diagnostic biomarkers of male infertility by specifically measuring serum levels of ER stress-related proteins. Although the biomarker potential of ER stress has been previously investigated in the literature, there is no comprehensive study on the role of these specific proteins in male infertility.

The proteins PDIA1, PDIA3, MANF, GRP78, CLU, CRT, and CNX were selected for this study due to their significant roles in the ERS response, which is closely linked to cellular homeostasis and survival, particularly in the context of male reproductive health. PDIA1 and PDIA3 are crucial for the formation of disulfide bonds during protein folding in the ER, playing a key role in maintaining protein homeostasis under stress, which is vital for sperm function. MANF is known for its protective effects against ER stress and its involvement in cellular recovery, potentially influencing sperm cell survival. GRP78, a master regulator of the UPR, is a key marker of ER stress and has a significant impact on spermatogenesis and sperm quality. CLU regulates apoptosis and stress responses, offering protection against ER stress-induced cell death. Finally, CRT and CNX are essential chaperones involved in the proper folding and quality control of glycoproteins in the ER, which is critical for the development and function of sperm. The inclusion of these proteins in the study aims to explore their potential as biomarkers for male infertility, given their established roles in ER stress and reproductive processes.

Alterations in ER homeostasis, or ERS, are directly linked to the pathophysiology of illnesses affecting reproduction.^42^ This preliminary investigation involved measuring and comparing the serum protein levels of PDIA1, PDIA3, MANF, GRP78, CLU, CRT, and CNX in patients diagnosed with idiopathic nonobstructive azoospermia and noninfertile male controls.

## Results

### Comparing infertile and noninfertile groups' serum levels of candidate biomarker proteins

We conducted a thorough literature review to identify proteins meeting our selection criteria. Based on this review, we selected PDIA1, PDIA3, MANF, GRP78, CLU, CRT, and CNX as chaperones for validation as serum-based biomarkers in potentially infertile populations. Several of these proteins have been reported in infertility.^32^, ^42^ It is noteworthy that CLU, typically secreted *via* the ER-Golgi secretory pathway, is redirected to the cytosol under ER stress conditions. There, it may play a role in trafficking misfolded proteins for degradation through the proteasome and/or autophagy pathways. These proteins have been previously detected in both blood and the central nervous system, indicating their potential for detection in serum samples.

**Fig. 1:**
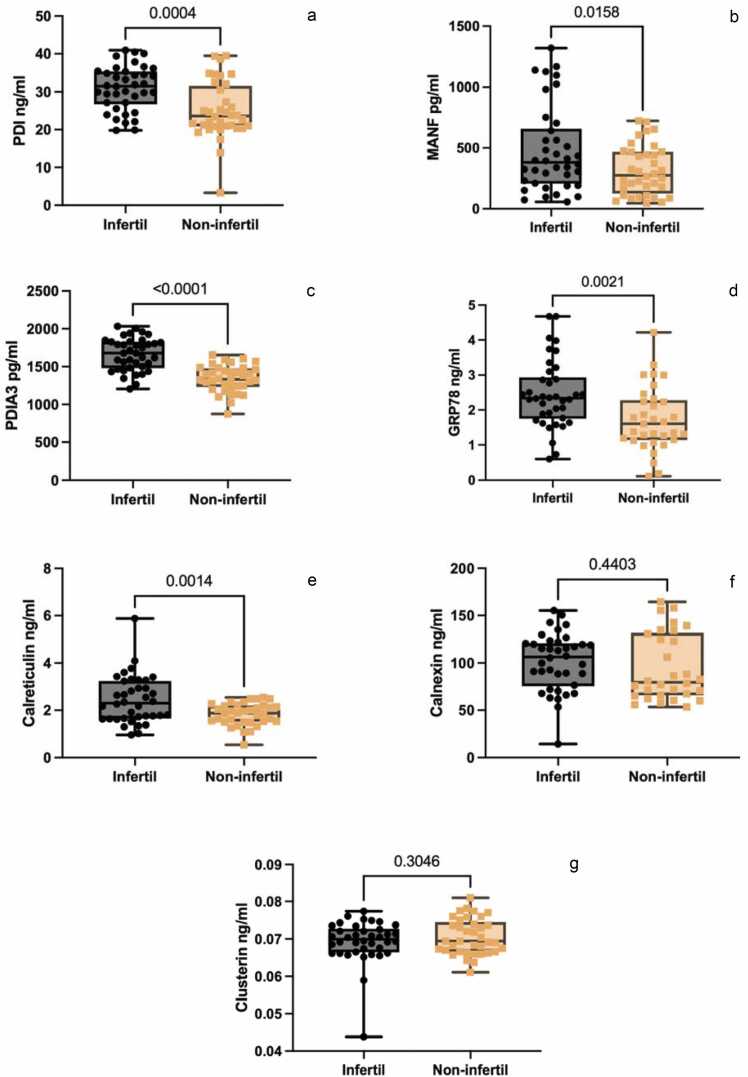
Serum concentrations of a PDIA3, b MANF, c PDIA3, d GRP78, e calreticulin, f calnexin, and g clusterin are graphically represented as individual values across the specified diagnostic groups. In box-and-whisker plots, the horizontal line within the box represents the median, while the lower and upper boundaries of the box indicate the 25th and 75th percentiles, respectively. Whiskers extend from the minimum value at the bottom to the maximum value at the top of the plot. Two-tailed t test with Welch’s correction was performed. Statistical significance was defined as *P*-values < 0.05. Abbreviations used: GRP78, glucose regulatory protein 78; MANF, mesencephalic astrocyte-derived neurotrophic factor; PDI, protein disulfide isomerase; PDIA3, protein disulfide-isomerase A3.

Enzyme-linked immunosorbent assay analysis was conducted to ascertain the concentrations of these proteins in the serum samples obtained from both infertile patients and noninfertile controls. This analysis revealed elevated levels of PDI (*P* = 0.0004) (Figure 1(a)), MANF (*P* = 0.018) (Figure 1(b)), PDIA3 (*P* < 0.0001) (Figure 1(c)), GRP78 (*P* = 0.0027) (Figure 1(d)), and CRT (*P* = 0.0009) (Figure 1(e)). Conversely, there was no significant difference observed in the levels of CNX (*P* = 0.4298) (Figure 1(f)) and CLU (*P* = 0.4430) (Figure 1(g)). Efforts to measure α-synuclein levels were unsuccessful due to them falling below the limit of detection (Table 3, Table 2).

**Table 1:** Age distribution between groups.

Group	Sample size (n)	Age (years), mean±SD
Infertile	38	36.11 ± 5.13
Noninfertile	36	41.14 ± 8.51

Abbreviation used: SD, standard deviation.

**Table 2:** Protein serum concentrations in the designated diagnostic groupings.

	Infertil (n = 38)	Noninfertil (n = 36)	*P*-value	Cohen's d	Eta squared
PDIA1 (ng/ml), mean ± SD, (95% CI)	30.94 ± 5.857, (26.75–35.31)	25.07 ± 7.568, (20.86–31.62)	0.0004	0.867	0.1734
MANF (pg/ml), mean ± SD, (95% CI)	482.0 ± 353.5, (206.4–657.8)	316.8 ± 202.4, (125.3–468.0)	0.0158	0.574	0.09393
PDIA3 (pg/ml), mean ± SD, (95% CI)	1663 ± 214.4, (1481–1827)	1342 ± 176.9, (1231–1477)	<0.0001	1.633	0.4127
GRP78 (ng/ml), mean ± SD, (95% CI)	2.44 ± 0.96, (1.75–2.93)	1.72 ± 0.92, (1.13–2.28)	0.0021	0.766	0.1302
Calreticulin (ng/ml), mean ± SD, (95% CI)	2.44 ± 1.00, (1.65–3.24)	1.83 ± 0.46, (1.51–2.20)	0.0014	0.784	0.1770
Calnexin (ng/ml), mean ± SD, (95% CI)	101.2 ± 30.29, (75.47–120.8)	94.89 ± 35.55, (65.76–131.7)	0.4403	0.191	0.01047
Clusterin (ng/ml), mean ± SD, (95% CI)	0.07 ± 0.01, (0.06–0.07)	0.07 ± 0.005, (0.066–0.074)	0.3046	0.000	0.01471

Abbreviations used: GRP78, 78-kDa glucose-regulated protein; MANF, mesencephalic astrocyte-derived neurotrophic factor; PDIA1, protein disulfide isomerase A1; PDIA3, protein disulfide isomerase A3; SD, standard deviation; Cl, Confidence level.

Nonetheless, there was a notable convergence in the measured protein levels within the diagnostic categories. The observed variations in mean protein levels between the diagnostic groups were statistically significant, notwithstanding this overlap. This indicates that five of the investigated proteins may hold promise in discerning between infertile and noninfertile groups.

**Fig. 2:**
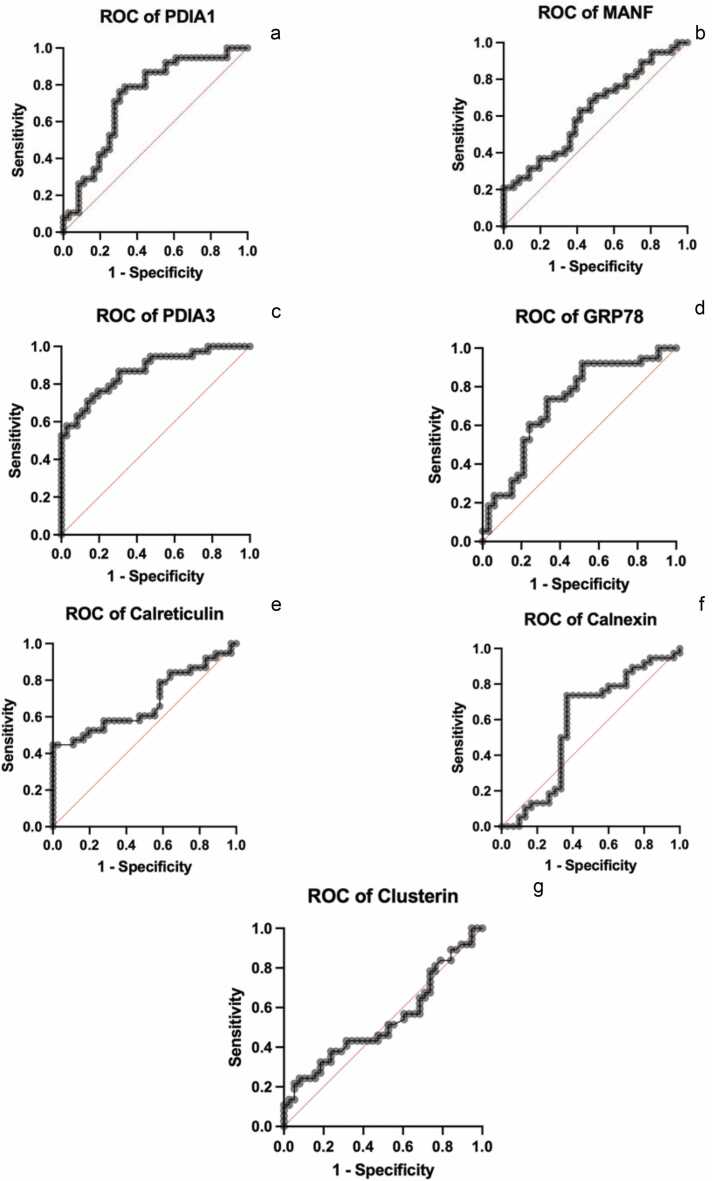
The ROC curves of serum PDIA3, MANF, PDIA3, GRP78, calreticulin, calnexin, and clusterin. Abbreviations used: GRP78, 78-kDa glucose-regulated protein; MANF, mesencephalic astrocyte-derived neurotrophic factor; PDI, protein disulfide isomerase; PDIA3, protein disulfide isomerase A3; ROC, receiver operating characteristics.

The results obtained from the ROC curve analysis reveal the discriminatory power of various biomarkers in the diagnosis of male infertility. Among the biomarkers investigated, PDIA3, MANF, PDIA3, GRP78, and CRT exhibit promising performance with area under the ROC curve values of 0.7303, 0.6279, 0.8699, 0.7161, and 0.6827, respectively. These values indicate that these biomarkers have a strong potential for distinguishing between fertile and infertile individuals. Furthermore, the area under the ROC curve values for CNX and CLU are 0.5781 and 0.5338, respectively. While these values are still above 0.5, suggesting some discriminatory ability, they are comparatively lower than those of the other biomarkers mentioned. Overall, biomarkers with higher area under the ROC curve values, closer to 1, are indicative of greater potential as diagnostic markers for male infertility. In this context, PDIA3, MANF, PDIA3, GRP78, and CRT emerge as particularly promising candidates for further investigation and potential clinical application in the diagnosis of male infertility (Figure 2).

### Differential expression analysis of target mRNAs using Gene Expression Omnibus database

The initial comparison of target mRNA expression profiles between infertile male and healthy male testicular tissue biopsy samples was conducted using publicly available data sets GSE145467, GSE45885, and GSE45887 obtained from the Gene Expression Omnibus (GEO) database. Detailed characteristics of the tissue biopsy samples can be found in the original data sources.^43^, ^44^, ^45^ The GEO2R analysis tool was employed for target mRNA expression analysis of infertile male and healthy control samples. In the analysis of the results from various GEO databases, it was observed that the mRNA expression levels of the human targets PDIA3, MANF, CLU, CRT, and CNX were decreased in the infertile male group compared to the control group, as reported in the respective data sets. However, upon conducting our analysis of human serum samples, distinct findings emerged. Specifically, we found that the levels of PDIA3, MANF, and CRT were increased in the infertile male group compared to the control group. Conversely, no significant difference was observed in the levels of CLU and CNX between the infertile male and control groups (Table 3).

**Table 3:** Gene expression changes of control *versus* infertile male patients’ testicular biopsy samples in three different public data sets.

Gene symbol	Gene titile	padj	pval.	LogFC	GEO data set
PDIA3	Protein disulfide-isomerase A3	6.62E−03	1.95E−03	−0.73466	GSE145467
		0.63563	3.59E−01	−0.227837	GSE45887
MANF	Mesencephalic astrocyte-derived neurotrophic factor	7.4E−01	6.5E−01	−0.12225	GSE145467
		7.88E−01	5.93E−01	0.05814	GSE45885
		0.9913	9.77E−01	−0.003693	GSE45887
CLU	Clusterin	8.72E−01	8.15E−01	−0.06387	GSE145467
		5.90E−01	3.37E−01	0.13885	GSE45885
		0.63488	3.58E−01	0.1493219	GSE45887
CRT	Calreticulin	9.6E−03	3.04E−03	−0.76207	GSE145467
		6.29E−01	3.80E−01	−0.11793	GSE45885
		0.68072	4.18E−01	−0.120197	GSE45887
CNX	Calnexin	2.3E−02	8.67E−03	−0.65066	GSE145467
		2.55E−02	2.08E−03	−0.32032	GSE45885
		0.15853	2.12E−02	−0.30164	GSE45887

Abbreviation used: GEO, Gene Expression Omnibus.

These contrasting results between testicular tissue biopsy samples and serum samples suggest potential differences in gene expression patterns between these sample types or highlight the complexity of gene expression regulation in the context of male infertility. Additional studies are warranted to elucidate the underlying mechanisms driving these observed differences and their implications for diagnosing and understanding male infertility.

## Discussion

Infertility is a health issue that leads to considerable psychological and social distress while also imposing a substantial economic burden on the healthcare system.^50^ Early diagnosis and proper referral can reduce the factors contributing to infertility. Severe male infertility has also been correlated with an increased incidence of cancer.^51^ Therefore, early detection of male infertility presents an opportunity to recognize and address medical conditions that affect not only fertility but also overall health and quality of life.^52^ Although there are recent studies on the role of ERS in the male reproductive system, specific studies in this field are limited.^53^

This preliminary investigation marks the initial effort to assess the efficiency of ER stress-regulated chaperone proteins, examined in serum, in distinguishing between infertile male patients and male controls without infertility. In this study, we identified ER stress-associated proteins present in blood, which demonstrate modified secretion during ER stress. Each of the seven proteins was individually detected in serum samples from infertile male patients and noninfertile male controls, with five out of these seven proteins enabling discrimination between infertile male patients and noninfertile male controls. Correct protein folding is crucial for proper protein function. PDI A1 serves as a vital chaperone, contributing to protein quality control and redox regulation. Within sperm physiology, various molecular chaperones, such as those belonging to the PDI family, play significant roles in ensuring precise protein folding and secretion.^54^ Additionally, it implies that PDIA1 might have implications in the process of epididymal maturation and male fertility. Research on animals has indicated that, during peripuberty, PDIA1 expression in the epididymis increases. However, its amount in adult animal seminal plasma was not reported to be associated with fertility.^33^ Nevertheless, serum levels of PDIA1 have not been previously documented in azoospermia. Our findings reveal elevated PDIA1 levels in the infertile group.

The MANF is a protein and secretory factor localized in the ER and has been implicated in many diseases.^55^ However, despite its documentation in various diseases, there has been no investigation into its levels concerning male infertility. Our findings demonstrate elevated MANF serum levels in infertile individuals, representing the first report of such data. PDIA3 was shown to be localized in the mouse sperm membrane, potentially forming a complex with lectin-like chaperones, and is suggested to play a crucial role in sperm–egg fusion.^37^ Subsequently, Maselli *et al.*^56^ reported PDIA3 on rat sperm head. Another study indicated that PDIA3 expression levels in human spermatozoa correlate with fertility. *In vitro* fertilization patients with low fertilization rates showed a notable decrease in semen PDIA3 protein expression compared to individuals with high rates or fertile sperm donors.^38^ In our study, PDIA3 serum levels were found to be higher in infertile patients. However, the scarcity of comprehensive data in the existing literature constrained our ability to engage in thorough discussion.

GRP78, commonly situated in the ER, serves a role in stabilizing ER proteins and triggering the UPR. Although also expressed on spermatozoa surfaces, its function in sperm physiology remains ambiguous. A study utilizing animal semen samples revealed that GRP78 significantly influences sperm function *via* the PI3K/PDK1/AKT pathway.^57^ Another study found reduced serum GRP78 levels in patients with asthenozoospermia compared to controls.^58^ Despite investigations into the impact of GRP78 protein on male infertility, its levels have not been reported in idiopathic nonobstructive azoospermia patient groups.^59^ In our findings, serum GRP78 levels were elevated in the infertile patient group compared to controls. CLU, a pivotal seminal plasma glycoprotein, plays crucial roles in sperm capacitation and immune tolerance, and it is also recognized as a sensitive marker of oxidative stress. In a study on CLU, seminal plasma levels were compared between nine azoospermic patients with successful micro-Testicular sperm extraction surgery and 19 patients for whom micro-Testicular sperm extraction surgery failed, revealing a statistically significant difference.^60^ However, in our results, no disparity was observed in serum CLU levels between the infertile patient group and controls. The areas under the curve and *P*-values calculated from the ROC curve analysis suggest that PDIA3, MANF, GRP78, and CRT could serve as potential biomarkers for diagnosing male infertility. However, the same level of confidence cannot be attributed to CNX and CLU. These findings indicate that the former set of markers demonstrates promising diagnostic potential, whereas the latter may not be as reliable in this context. Further investigation is necessary to validate these results and ascertain the clinical significance of the identified biomarkers in male infertility diagnosis.

While mRNA expression levels are commonly used as proxies to predict functional differences at the protein level, the relationship between mRNA and protein expression remains inadequately established. It is often assumed that many differences in mRNA expression may not be reflected at the protein level, or *vice versa*. According to the results obtained from the GEO database, the mRNA levels of human PDIA3, MANF, and CRT exhibit discrepancies when compared to serum protein levels. Additionally, although the mRNA levels of the target genes CLU and CNX decrease in the male infertile group compared to the control group, the serum levels of CLU and CNX do not differ between the control and infertile groups. This discrepancy indicates a divergence between mRNA expression and actual protein expression. Previous studies have documented instances of negative correlation between mRNA levels and actual protein levels.^61^, ^62^

Our study presents a panel of ER stress-regulated chaperones investigated within a cohort of patients diagnosed with idiopathic nonobstructive azoospermia. These findings provide valuable initial insights into the diagnosis and monitoring of azoospermia. However, it is crucial to recognize the limitations of our study. Initially, our research was confined to the Turkish population, leaving uncertainty regarding the generalizability of the results to other populations. Furthermore, prior reports on serum levels of ER stress-regulated chaperones in azoospermia were lacking, posing challenges during the discussion phase. Therefore, additional functional investigations and larger sample sizes are warranted to thoroughly understand the involvement of ER stress-regulated chaperones in the diagnosis and pathogenesis of azoospermia.

In this study, we assessed the potential of ER stress-related chaperone proteins as biomarkers for male infertility by analyzing Cohen's d effect sizes. The Cohen's d values for markers such as PDIA3, MANF, and GRP78 were 1.633, 0.574, and 0.766, respectively, strongly supporting the association of these proteins with infertility. The particularly high effect size for PDIA3 suggests that this protein could be a significant biomarker in cases of infertility. In contrast, the low Cohen's d values obtained for CNX and CLU (0.191 and 0.000, respectively) indicate a weaker relationship or suggest that these proteins may not have a significant impact in this specific sample. These findings underscore the need for further investigation into the potential value of specific chaperone proteins as biomarkers for infertility.

The discussion provides a thorough analysis of the findings, but it could be enhanced by addressing potential limitations and suggesting directions for future research. One significant limitation to consider is the sample size, which, although sufficient for preliminary conclusions, may not fully represent the broader population. Additionally, the potential influence of confounding factors, such as age, lifestyle, and underlying health conditions, should be discussed, as these could have affected the outcomes. Furthermore, it is important to acknowledge any methodological constraints, such as the reliance on specific enzyme-linked immunosorbent assay protocols, which might limit the generalizability of the results. Future studies could benefit from larger, more diverse samples and should consider alternative or complementary methodologies to validate these findings and expand our understanding of ER stress-related chaperones in male infertility.

In summary, our study presents pioneering data on ER stress-regulated chaperone proteins within an azoospermia patient cohort. This pilot study suggests that further validation of these proteins in a larger cohort could enhance diagnostic accuracy. Evaluating the model across various male infertility disorders and diverse patient cohorts representing ER stress-related disorders will be crucial. Additionally, our results underscore the potential benefits of incorporating additional biomarkers into the panel to improve diagnostic performance. This paper highlights the potential of ER stress-regulated proteins as serum-based biomarkers for azoospermia and highlights the importance of integrating different biomarkers.

Understanding the role of ER stress-regulated proteins in male infertility could lead to the development of novel diagnostic tools and therapeutic strategies for azoospermia. By targeting ER stress pathways, clinicians may be able to intervene early in the disease process and improve outcomes for infertile patients. Additionally, the identification of serum-based biomarkers could facilitate noninvasive diagnostic testing and monitoring of male infertility, enhancing clinical management and patient care. Further research in this area is warranted to fully elucidate the clinical significance of ER stress-regulated proteins in male reproductive health.

## Conclusion

In conclusion, our pilot study indicates elevated serum concentrations of PDIA1, PDIA3, MANF, GRP78, CLU, and CRT in male patients diagnosed with idiopathic nonobstructive azoospermia, as compared to noninfertile control subjects. This suggests a potential association between ER stress-regulated proteins and male infertility. The identification of these biomarkers may provide valuable insights into the underlying mechanisms contributing to azoospermia and might facilitate the development of targeted therapeutic interventions. To validate these results and determine the clinical value of ER stress-regulated proteins as diagnostic or prognostic indicators for male reproductive diseases, more investigation is necessary.

## Materials and methods

### Participant recruitment and evaluation

The cross-sectional pilot project was authorized by the Clinical Research Ethics Committee of Yuksek Ihtisas University (protocol number: 2023-02), and subjects provided written informed consent. The patient group consisted of men with diagnosed idiopathic nonobstructive azoospermia, whereas the healthy control group consisted of men who were not diagnosed with infertility. Healthy controls were selected from individuals with known fertility history and normal sperm analysis results. Inclusion criteria: As inclusion criteria, only male participants aged 18–50 years were included in the study. For the infertile group, patients were required to have been unable to have children despite unprotected sexual intercourse for at least 1 year, and no obstructive cause was found in the examinations. In the healthy control group, men with normal sperm parameters were selected. Exclusion criteria: Exclusion criteria were defined as any urogenital infection, systemic disease (e.g., diabetes, heart disease), hormone therapy, or previous surgical intervention on the reproductive system. Factors such as smoking, alcohol, or drug use were also excluded. These criteria were set to ensure that the results were only relevant to infertility.

A power calculation was conducted to determine the sample size required for detecting a difference between male infertility and male noninfertility (control) groups, and the sample size was calculated to be a minimum of 36 patients. This calculation was performed with a power level of 80%, an alpha error of 5%, and an effect size of 0.6 using G*Power 3.1 software developed by Heinrich Heine University, Düsseldorf, Germany. Blood samples were collected from 38 infertile men (with idiopathic nonabstructive azoospermia) and 36 healthy men. The 38 infertile male patients who participated in the study were diagnosed with idiopathic nonabstructive azoospermia. Table 1 shows the age distribution of the enrolled individuals.

### Collection and processing of human serum samples

The venous blood collection procedure involved the use of BD Vacutainer serum tubes (Becton, Dickinson, NJ, USA; #367953), which contain silica for promoting clot formation. This process was administered by a trained phlebotomist. Following collection, the samples were left to clot in a dark environment at room temperature for a duration of 1 h. Subsequently, the tubes underwent centrifugation at 1300× *g* for 10 min. The resulting serum was carefully extracted and divided into polypropylene 0.5 ml tubes. Within 50 min post centrifugation, the serum samples were promptly stored at -80°C to maintain their integrity for subsequent analysis.

### Enzyme-linked immunosorbent assay

Serum levels of PDI (fine test; EH3539), MANF (fine test; EH3322), PDIA3 (fine test; EH3541), GRP78 (fine test; EH1801), CRT (fine test; EH1659), CNX (fine test; EH2856), CLU (fine test; EH0004), and α-synuclein (fine test; EH0582) were determined using enzyme-linked immunosorbent assays following the manufacturer's protocols. These assays utilized a sandwich enzyme-linked immunosorbent assay technology. The 96-well plates were precoated with anti-PDI, anti-MANF, anti-PDIA3, anti-GRP78, anti-CRT, anti-CLU, anti-α-synuclein, and anti-CNX antibodies. Biotin-conjugated antiantibodies were employed for detection. After that, the wells were filled with standards and pilot samples. Wash buffer was used to eliminate unattached conjugates following incubation. Biotinylated detection antibodies were added to bind to the target proteins. Horseradish peroxidase-streptavidin was added after unbound conjugates were washed away. Tetramethylbenzidine substrates were added during the third wash in order to observe the Horseradish peroxidase enzymatic response. When the stop solution was added, the blue product that Tetramethylbenzidine, catalyzed by Horseradish peroxidase, had created turned yellow. The absorbance at 450 nm was measured using a microplate reader. Protein concentrations in the samples were determined by plotting a standard curve, where the concentration of the target substance was directly proportional to the OD450 value. To increase the reliability of enzyme-linked immunosorbent assay results, each sample was tested in duplicate and averaged.

### Differential expression analysis of target mRNAs through GEO2R

The Gene Expression Omnibus (https://www.ncbi.nlm.nih.gov/gds) database was used for mRNA expression profiling studies in infertile male and control male testicular biopsy samples. The GEO database is a public platform maintained by the National Center for Biotechnology Information. It hosts a vast collection of high-throughput gene expression data, including microarray and next-generation sequencing data, from a wide range of organisms and experimental conditions. Target mRNAs are differentially expressed between infertile male testicular biopsy specimens, and healthy male testicular biopsy specimens were identified by GEO2R from the GSE145467, GSE45885, and GSE45887 data sets (accessed on April 24, 2024). GSE145467 data set was obtained from a study that performed gene expression analysis using Agilent-014850 Whole Human Genome Microarray 4x44K G4112F,^43^ GSE45885, and GSE45887 data sets was obtained from studies that performed Affymetrix Human Gene 1.0 ST array.^44^, ^45^

### Statistical analysis

In assessing the data's normality, particularly considering the sample size (<50 samples), the Shapiro–Wilk test,^46^, ^47^ was employed. Normal distribution was observed in all data sets, addressed using unpaired two-tailed t test with Welch’s correction. Statistical significance was defined as *P*-values < 0.05. All statistical analyses were carried out with GraphPad Prism Software Version 9.0 (San Diego, CA, USA).

Prediction models are commonly evaluated by applying them to a data set and comparing their predictions with actual patient outcomes. Usually, measurements like the area under the ROC curve are used to evaluate these models' performance. The area under the curve, as described by Sakurai *et al.*,^48^ can be interpreted as the probability that, in a pair of individuals, one who experienced the event and one who did not, the individual who experienced the event has a higher predicted probability.

Upon obtaining results, we constructed the ROC curve and computed the corresponding areas under the curve. A larger Area under the curve, exceeding 0.5, indicates a higher discriminative ability of the model.^49^

## Ethics statement

This study was approved by the Clinical Research Ethics Committee of Yuksek Ihtisas University (protocol number: 2023-02).

## Funding and support

This work was funded from Yuksek Ihtisas University (grant no.: 2023/01.015) to CC.

## Author contributions

**Osman Denizhan Ozgun** and **Cumhur Bilgi:** Investigation. **Semra Sertyel:** Supervision, Resources, Methodology, Investigation. **Pelin Telkoparan-Akillilar:** Writing – review & editing, Writing – original draft, Methodology, Investigation, Conceptualization. **Cigdem Cicek:** Writing – review & editing, Writing – original draft, Project administration, Funding acquisition.

## Declarations of interest

The authors declare that they have no known competing financial interests or personal relationships that could have appeared to influence the work reported in this paper.

## Data Availability

Data will be made available on request.
